# Fertility and pregnancy outcomes following hysteroscopic metroplasty of different sized uterine septa: A retrospective cohort study protocol

**DOI:** 10.1097/MD.0000000000016623

**Published:** 2019-07-26

**Authors:** Xi Wang, Haiyan Hou, Qi Yu

**Affiliations:** a Department of Obstetrics and Gynecology, Peking Union Medical College Hospital, Peking Union Medical College and Chinese Academy of Medical Sciences; b Department of Obstetrics and Gynecology, Peking University First Hospital; c Department of Obstetrics and Gynecology, Affiliated Hospital of the Chinese People's Armed Police Force Logistics College, Tianjin, China.

**Keywords:** fertility, pregnancy, uterine septum

## Abstract

Different sizes of uterine septum between infertile women and patients with abortions may have a clinical relevance in reproductive performance after surgery. This study aimed to assess if the fecundity of women after surgical correction of the uterine septum is associated with septum size.

A retrospective, single-center, cohort study was conducted in Peking Union Medical College Hospital using patients aged between 21 and 37 years. Hysteroscopic metroplasty was performed on 121 patients with a uterine septum. The septum size was assessed by ultrasonography and hysteroscopy. The subjects were divided into 3 groups: Group A consisted of 35 women with complete uterine septum (mean ± standard deviation (SD) age 28.29 ± 3.53; group B consisted of 48 women with uterine septum >2.5 cm (mean ± SD age 28.85 ± 3.63); and group C consisted of 48 women with uterine septum ≤2.5 cm (mean ± SD age 28.79 ± 3.74). Age and body mass index (BMI) were not significantly different among the 3 groups.

No serious hysteroscopic complications occurred. However, uterine septa were observed in 4 cases after surgery and 6 cases of intrauterine adhesions were observed after long-term follow-up. The abortion rate decreased, and term delivery rate increased significantly in the 3 groups after hysteroscopic metroplasty. The infertility rate was significantly lower in group C after surgery. However, no significant difference was observed in the infertility rate between groups A and B. The recurrent abortion rate was significantly lower in group A than in groups B and C before surgery. After surgery, the infertility rate was significantly higher in group A than in group B (28.57% and 10.53%, respectively; *P* = .048). After at least 12-months of follow-up, the pregnancy rate in group A was significantly lower than that in group C (71.43% and 89.47%, respectively; *P* = .048).

Uterine septum resection improves obstetrical outcomes. After surgery, the infertility rate was significantly higher in patients with complete uterine septum than in those with a large partial uterine septum, and the pregnancy rate in patients with complete uterine septum was lower than that in the patients with a small partial uterine septum.

## 1 Introduction

Septate uterus is a congenital anomaly characterized by the persistence of the partition resulting from a defect of fusion of paramesonephric ducts during embryogenesis. Many studies have reported that abnormalities of the uterine septum are associated with reproductive failures, such as miscarriage, recurrent pregnancy loss, and obstetric complications, including recurrent abortions of the first and second trimester, intrauterine growth retardation, and abnormal fetal presentation. The true prevalence of the uterine septum is difficult to affirm as many uterine septum defects are asymptomatic but appear to range from 1 to 2 per 1000 to as high as 15 per 1000 individuals.^[1]^ The mean prevalence of uterine malformations in the general population of fertile women is about 4.3%, in infertile patients, about 3.5% and in patients with recurrent pregnancy loss is 5 to 25%,^[2–4]^ with septate uteri being the most frequent, at an incidence of 5.3%.^[5]^ According to the most accepted classification system of the American Society for Reproductive Medicine (ASRM) worldwide,^[6]^ an arcuate uterus corresponds to Class VI, complete uterine septum to Class Va, and partial septate uterus to Class Vb. Some studies have indicated that hysteroscopic metroplasty of uterine abnormalities can improve pregnancy outcomes in women with a history of recurrent pregnancy loss.^[7,8]^ A retrospective matched-control study evaluated the effect of uterine anomalies on pregnancy rates after 2481 embryo transfers in conventionally stimulated in-vitro fertilization (IVF)/intracytoplasmic sperm injection (ICSI) cycles. The study groups of 289 embryo transfers before and 538 transfers following metroplasty were compared with 2 consecutive embryo transfers in the control group. The pregnancy rate before hysteroscopic resection of the uterine septum was lower than that after hysteroscopic resection, and the abortion rate was higher. The results showed that the presence of septate uterus decreased the pregnancy rate and increased the abortion rate after embryo transfers for IVF/ICSI.^[9]^ However, these findings have not been verified in a prospective, randomized, controlled trial (RCT) comparing different treatments of the uterine septum to without treatment. To date, there is limited data reported on the impact of different septum sizes within Class Va and Vb on reproductive performance.

The aim of the study was to compare the reproductive outcomes of pregnancies in the same women before and after hysteroscopic resections of the uterine septum according to septum size. We evaluated, if different sizes of the uterine septum between infertile women and in patients with earlier abortions may have clinical relevance in reproductive performance after surgery.

## 2 Methods

### 2.1 Ethical approval

The study was approved by the Institutional Review Board (IRB) of Peking Union Medical College Hospital (number: S-K511). Informed consent was obtained in accordance with the institutional guidelines.

### 2.2 Study design and population

This was a single center, retrospective analysis of subjects at Peking Union Medical College Hospital from a population of uterine septum patients aged between 21 and 37 years referred to the outpatient infertility clinics of our institutions. Only women with body mass indexes (BMIs) ranging between 18.5 and 28 kg/m^2^, were considered. Small and large partial uterine septa were defined as those with a length of ≤2.5 cm, and not complete, with a length of ≥2.5 cm, respectively.^[10,11]^ All septa were classified according to the ASRM classification: 35 patients had a complete uterine septum (class Va) representing group A, 48 had a large partial uterine septum (class Vb) representing group B, and 38 had a small partial uterine septum (class Vb) representing group C. Patients with pelvic lesions, such as endometriosis; oligo- or anovulation and menstrual irregularities; partners with abnormal semen analysis, which could adversely affect fertility were excluded from the study. The primary criteria for inclusion were accurate data on the size of the septum, and patients with a history of pregnancy loss or infertility. Of 279 treated patients, only a cohort of 121 women, operated between July 2006 and January 2017, fulfilled these requirements, based on records of office hysteroscopy with laparoscopy available in most cases. Accordingly, the subjects were divided into 3 groups. Group A consisted of 35 women with complete uterine septum (mean ± standard deviation (SD) age 28.29 ± 3.53 95% confidence interval (CI) 27.07–29.50]; BMI 21.69 ± 2.92 [95% CI 20.67–22.71]); group B consisted of 48 women with uterine septum >2.5 cm (mean ± SD age 28.85 ± 3.63, [95% CI 27.80–29.91]; BMI 22.13 ± 2.94 [95% CI 27.80–29.91]); and group C consisted of 38 women with uterine septum ≤2.5 cm (mean ± SD age 28.79 ± 3.74, [95% CI 27.56–30.02]; BMI 21.97 ± 2.94 [95% CI 22.13–23.81]). Age and BMI were not significantly different among the 3 groups (*P* = .76, *P* = .59) (Table 1).

**Table 1:**
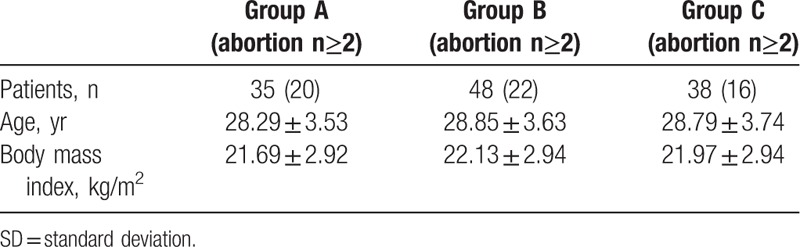
Patient characteristics.

We additionally assessed the reproductive outcomes at ≥1 year of trying to get pregnant after surgery and compared them with those based on previous obstetric history. This period was selected because, by definition, a patient should be defined infertile if they fail to achieve a clinical pregnancy after 12 months or more of regular unprotected sexual intercourse. The patients were then divided into 2 sub-groups according to their fertility history: 48 infertile patients and 86 aborters. Fourteen patients who had had an abortion, and were unable to get pregnant for at least 1 year were diagnosed with secondary infertility before the preoperative follow-up.

### 2.3 Operative procedure

It was documented in the patient records that the details of the operation and potential complications were explained to each patient. Each woman was advised to undergo hysteroscopic resection. The surgery of hysteroscopic metroplasty was performed in the early proliferative phase in the operating room under general anesthesia. After the cervix was dilated to 10 mm, a 7 mm rigid hysteroscope (model 26050 EG; Karl Storz, Tuttlingen, Germany) was introduced into the cervix. Five percent dextrose or mannitol was used to distend the uterine cavity, and the uterine distention pressure was set at about 150 mm Hg. The septum was dissected using a continuous flow resectoscope (Karl Storz, Tuttlingen, Germany). Electrosurgical incision in the uterine septum was made equidistantly between the anterior and posterior uterine walls and went up high into the uterine fundus until the presence of a normal-shaped cavity was obtained. A combination of estrogens and intrauterine device (IUD) was used to prevent adhesions.

Patients were advised to have an ultrasonographic and hysteroscopic examination after 3 to 4 months to confirm surgical outcomes and evaluate the presence of residual uterine septa or intrauterine adhesions. Follow-up was performed through a telephone conversation with particular emphasis on the desire to become pregnant, pregnancy status, and outcomes. A call was placed at least 6 times on different days before confirming the loss of follow-up. A total of 360 patients were followed up and 81 were lost during follow-up due to a wrong phone number or refusal to answer questions.

### 2.4 Statistical analysis

The mean ± (standard deviation) SD and 95% CI were used to report continuous data. The data were analyzed using the R (R i386 3.4.3.lnk, Bell Laboratory, NJ), and figures were prepared using GraphPad Prism 7 (GraphPad Software, La Jolla, CA). The paired *t* test was used to compare the age and BMI of the 3 groups. The paired data of pre- and post-operative reproductive outcomes were compared using Mc Nemar test. Z-testing was used to make 2 comparisons between the 3 groups in terms of reproductive outcomes before and after surgery, respectively. *P* <.05 was considered to be statistically significant.

## 3 Results

No serious hysteroscopic complications such as fluid overload, uterine perforation, or massive hemorrhage occurred. However, regarding long-term complications, residual uterine septa were observed in 4 cases after surgery, 3 of which were large partial uterine septa, and 1 was a complete uterine septum. Three patients with residual uterine septa achieved term deliveries, and 1 patient had an early miscarriage, refusing further surgery. There were 6 cases of intrauterine adhesions, 2 each belonging to groups A, B, and C. All patients suffering from intrauterine adhesions underwent a second operation to perform adhesion lysis. Finally, 2 patients achieved term deliveries, 1 patient gave a preterm live birth, 2 patients had an abortion, and 1 patient suffered from infertility. There were 4 cases of placental previa after the treatment of 3 large uterine septa cases and 1 small uterine septum. There was 1 case of heavy hemorrhage in group A.

In the present study, the reproductive history and performance before and after septum resection were analyzed in the 3 groups of patients (Tables 2–4). The abortion rate of the 3 groups decreased significantly (*P* <.05) and term deliveries rate increased significantly after the surgery. The infertility rate was significantly lower in group C after surgery (39.47% vs 10.53%, *P* = .006). However, no significant difference was observed in the infertility rate between groups A and B. There were no significant differences in pregnancy rate, ectopic pregnancy rate, and preterm live births rate among the 3 groups (*P* >.05 for all).

**Table 2:**
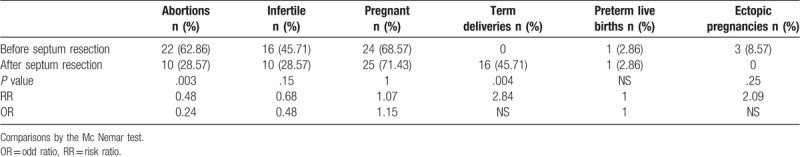
Comparison of pre- and post-operative outcomes in group A.

**Table 3:**
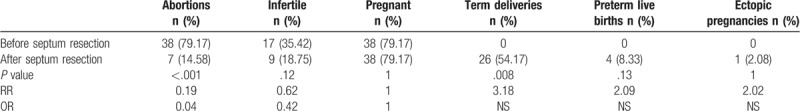
Comparison of pre- and post-operative outcomes in group B.

**Table 4:**
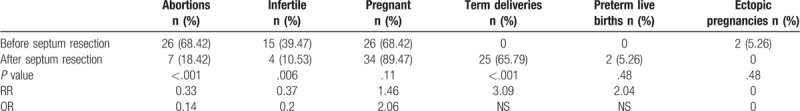
Comparison of pre- and post-operative outcomes in group C.

**Figure 1:**
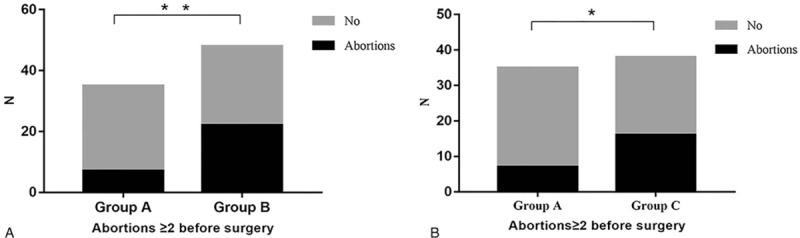
The abortion rate (n≥2) before hysteroscopic metroplasty of the uterine septum. The abortion rate (n≥2) was 20% (7/35) in group A, higher than that in groups B and C at 45.83% (22/48), and 42.11% (16/38) (^∗∗^*P* = .009,^∗^*P* = .03), respectively.

**Figure 2:**
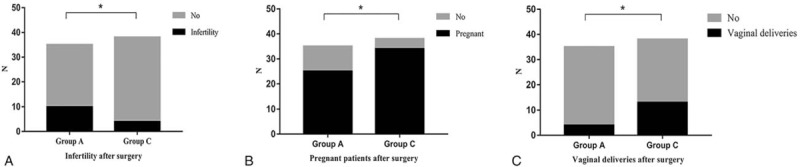
The infertility, pregnancy, and vaginal delivery rates after hysteroscopic metroplasty of the uterine septum. After surgery, the infertility rate was significantly higher in group A than in group B (28.57% and 10.53%, respectively; *P* = .048). After at least 12-months of follow-up, the pregnancy rate in group A was significantly lower than that in group C (71.43% and 89.47%, respectively; ^∗^*P* = .048). The vaginal delivery rate was 11.43% in group A, and 34.21% in group C (^∗^*P* = .01).

The main analyzed variables (infertility, pregnancy, abortion, term delivery rates, etc) before and after surgery are reported in Figures 1 and 2. The abortion rate (n ≥2) was 20% (7/35) in group A, lower than that in groups B and C with 45.83% (22/48), 42.11% (16/38) (*P* = .009, *P* = .03), respectively. There were no significant differences in other obstetrics history variables among the 3 groups before surgery (Table 2). After surgery, the infertility rate was significantly higher in group A than in group B (28.57% and 10.53%, respectively; *P* = .048). After at least 12-months of follow-up, the pregnancy rate in group A was significantly lower than that in group C (71.43% and 89.47%, respectively; *P* = .048). The vaginal delivery rate was 11.43% in group A, compared to 34.21% in group C (*P* = .01). There were no significant differences with respect to other reproductive performance variables among the 3 groups.

## 4 Discussion

Our study shows an excellent prognosis for a successful pregnancy after hysteroscopic metroplasty because septum resection highly improved reproductive performance in the 3 groups of patients, increasing delivery rates and indirectly decreasing abortion rates. Although it is known that a uterine septum leads to pregnancy losses, the relationship between uterine septum and infertility is still questionable. Uterine septum is a common uterine malformation leading to adverse reproductive outcomes; hysteroscopic septum resection improves the pregnancy outcomes significantly.^[9,11]^ It is known that the sensitivity of the endometrium to pre-ovulatory hormonal changes in the septal tissue is lower than that in the normal uterine tissue.^[12]^ Furthermore, Raga F et al reported that the number of vascular endothelial growth factor (VEGF) receptors in the transmembrane is significantly reduced in the septum endometrium, compared with that of the normal uterus.^[13]^ Therefore, uterine septum has been associated with poor obstetric outcomes. Hysteroscopic septum resection in women with a septate uterus is performed worldwide to improve reproductive outcomes. However, at present, there is no evidence to support the use of this surgical procedure in these women, and randomized controlled trials are urgently needed.^[14]^ Several studies ^[7,11,15,16]^ reported an increase in pregnancy rate after metroplasty in groups of infertile patients, ranging from 23% to 80.6%. Unfortunately, low numbers of patient, heterogeneity, and retrospective design, as well as the absence of a control group make the results of these studies hard to interpret. In literature, the impact of different septum size on the pregnancy outcome was rarely studied. Our data confirmed the efficacy of surgical treatment in infertile patients with small partial uterine septum after surgery. Some retrospective studies have shown that a small partial septum increases the risk of spontaneous abortions.^[17–19]^ Ban-Frangez H, et al found that compared with women with a normal uterus, the abortion rate per pregnancy after IVF or ICSI before hysteroscopic metroplasty was significantly higher in both women with a large (OR 25) and women with a small partial (OR12) septum. After surgery, the abortion rate decreased and was comparable to the abortion rate after IVF or ICSI in women with a normal uterus.^[20]^ It is interesting that no significant difference was observed in the infertility rate between patients with a complete and large partial uterine septum in our study. Data from assisted reproductive technologies (ART) have also provided different information in this respect. In a retrospective matched control study comparing 289 embryo transfers before and 538 transfers following hysteroscopic resection of a uterine septum to 2 consecutive embryo transfers in the control group, Tomazevicet al ^[9]^ found that pregnancy rates after embryo transfer and before hysteroscopic metroplasty were significantly lower, both in women with sub-septate and septate uteri and in women with arcuate uterus compared to controls. In contrast, Marcus et al^[21]^ found implantation and pregnancy rates similar to those of the general sterile population undergoing ART.

More recently, Ono et al^[7]^ published a retrospective, single-center, cohort study including 31 patients with a history of at least 2 miscarriages due to a uterine septum who underwent a hysteroscopic resection. In this study, the postoperative pregnancy rate was 83.9%, and at 1 year postoperatively, 5 women remained persistent infertile. When the infertile group was compared with the pregnancy group, women in the postoperative infertility group were significantly older than those in the pregnancy group; a multivariate analysis was used to identify the independent risk factors, which showed that age was an independent risk factor for persistent infertility.

The major finding of our study is that the reproductive history and obstetrical outcomes before and after hysteroscopic metroplasty were influenced by uterine septum size, which has been poorly and rarely studied previously. A threshold of approximately 2.5 cm was chosen based on the size of the uterine cavity, considering that uterine corpus without cervix has a length of approximately 5 cm.^[10,11]^ Our data were in agreement with findings in a recent study by Paradisi R,^[11]^ that evaluated 2 groups of patients with small and large partial uterine septum with respect to reproductive history and performance before and after surgery. No significant differences in reproductive performance were evident between patients with small and large partial uterine septa. However, we found that the recurrent abortion rate (n ≥2) was lower in complete uterine septum compared with small partial uterine septum before surgery. Various retrospective studies have generated data showing that a small partial septum increases the risk of spontaneous abortions. After surgery, the infertility rate was significantly higher in women with complete uterine septum than in those with a large partial uterine septum. After at least a 12-month follow-up period, the vaginal delivery and pregnancy rates in group A were lower than those in group C. In general, complete uterine septum has relatively poor reproductive outcomes after hysteroscopic metroplasty. The prognosis of complete uterine septum was relatively worse than partial uterine septum after surgery. Maybe it is associated with the healing area of different sizes of uterine septum.

Some noted limitations of this study include its retrospective design, the absence of a control group, and the relatively small sample size.

In conclusion, our findings suggest that uterine septum resection is an effective procedure that improves obstetrical outcomes; therefore, it should be recommended to women with a septate uterus who have undergone an abortion. After surgery, the infertility rate was significantly higher in women with a complete uterine septum than in those with a large partial uterine septum, and the pregnancy rate in patients with a complete uterine septum was lower than that in the patients with a small partial uterine septum.

## Acknowledgments

We are grateful to all the patients and the families in this study for their collaboration. The professional English language service “editage” was utilized in the production of this manuscript.

## Author contributions

**Conceptualization:** Xi Wang.

**Data curation:** Xi Wang.

**Formal analysis:** Haiyan Hou.

**Investigation:** Qi Yu.

**Methodology:** Xi Wang, Haiyan Hou.

**Software:** Xi Wang, Haiyan Hou, Qi Yu.

**Validation:** Haiyan Hou.

**Visualization:** Haiyan Hou.

**Writing – original draft:** Xi Wang.

**Writing – review & editing:** Xi Wang, Qi Yu.

## References

[1] Valle RF Ekpo GE Hysteroscopicmetroplasty for the septate uterus: review and meta-analysis. J Min Invas Gynecol 2013;20:22–42. 10.1016/j.jmig.2012.09.010 23312243

[2] Acien P Incidence of Mullerian defects in fertile and infertile women. Hum Reprod 1997;12:1372–6. 9262259 10.1093/oxfordjournals.humrep.a019588

[3] Pellicer A Shall we operate on Mullerian defects? An introductionto the debate. Hum Reprod 1997;12:1371–2. 9262258 10.1093/humrep/12.7.1371

[4] Grimbizis GF Camus M Tarlatzis BC Clinical implications of uterine malformations and hysteroscopic treatment results. Human Reprod Update 2001;7:161–74. 10.1093/humupd/7.2.161 11284660

[5] Chan YY Jayaprakasan K Zamora J The prevalence of congenital uterine anomalies in unselected and high-risk populations: a systematic review. Hum Reprod 2011;17:761–71. 10.1093/humupd/dmr028 PMC3191936 21705770

[6] The American Fertility Society. The American Fertility Society Classifications of Adnexal Adhesions, distal tubal occlusion, tubal occlusion secondary to tubal ligation, tubal pregnancies, Müllerian anomalies and intrauterine adhesions. Fertil Steril 1988;49:944–55. 3371491 10.1016/s0015-0282(16)59942-7

[7] Ono S Yonezawa M Watanabe K Retrospective cohort study of the risk factors for secondary infertility following hysteroscopicmetroplasty of the uterine septum in women with recurrent pregnancy loss. Reprod Med Biol 2017;17:77–81. 29371825 10.1002/rmb2.12072 PMC5768972

[8] Mollo A De Franciscis P Colacurci N Hysteroscopic resection of the septum improves the pregnancy rate of women with unexplained infertility: a prospective controlled trial. Fertil Steril 2009;91:2628–31. 18571168 10.1016/j.fertnstert.2008.04.011

[9] Tomazevic T Ban-Frangez H Virant-Klun I Septate, subseptate and arcuate uterus decrease pregnancy and live birth rates in IVF/ICSI. Reprod Biomed Online 2010;21:700–5. 20864409 10.1016/j.rbmo.2010.06.028

[10] Merz E Miric-Tesanic D Bahlmann F Sonographic size of uterus and ovaries in pre- and postmenopausal women. Ultrasound Obstet Gynecol 1996;7:38–42. 8932630 10.1046/j.1469-0705.1996.07010038.x

[11] Paradisi R Barzanti R Natali F Hysteroscopic metroplasty: reproductive outcome in relation to septum size. Arch Gynecol Obstet 2014;289:671–6. 24026089 10.1007/s00404-013-3003-9

[12] Fedele L Bianchi S Marchini M Ultrastructural aspects of endometrium in infertile women with septate uterus. Fertil Steril 1996;65:750–2. 8654633

[13] Raga F Casan EM Bonilla-Musoles F Expression of vascular endometrium of septate uterus. Fertil Steril 2009;92:1085–90. 19200976 10.1016/j.fertnstert.2008.07.1768

[14] Rikken JF Kowalik CR Emanuel MH Septum resection for women of reproductive age with a septate uterus. Cochrane Database Syst Rev 2017;11:CD008576. 10.1002/14651858.CD008576.pub4 PMC6464821 28093720

[15] Fayez JA Comparison between abdominal and hysteroscopicmetroplasty. Obstet Gynecol 1986;68:399–403. 2942813 10.1097/00006250-198609000-00023

[16] Shahrokh Tehraninejad E Ghaffari F Jahangiri N Reproductive outcome following hysteroscopic monopolar metroplasty: an analysis of 203 cases. Int J Fertil Steril 2013;7:175–80. 24520483 PMC3914491

[17] Salim R Regan L Woelfer B A comparative study of the morphology of congenital uterine anomalies in women with and without a history of recurrent first trimester miscarriage. Hum Reprod 2003;18:162–6. 12525460 10.1093/humrep/deg030

[18] Woelfer B Salim R Banerjee S Reproductive outcomes in women with congenital uterine anomalies detected by three dimensional ultrasound screening. Obstet Gynecol 2001;98:1099–103. 11755560 10.1016/s0029-7844(01)01599-x

[19] Makino T Hara T Oka C Survey of 1120 Japanese women with a history of recurrent spontaneous abortions. Eur J Obstet Gynecol Reprod Biol 1992;44:123–30. 1587377 10.1016/0028-2243(92)90057-6

[20] Ban-Frangez H Tomazevic T Virant-Klun I The outcome of singleton pregnancies after IVF/ICSI in women before and after hysteroscopic resection of a uterine septum compared to normal controls. Eur J Obstet Gynecol Reprod Biol 2009;146:184–7. 18524455 10.1016/j.ejogrb.2008.04.010

[21] Marcus S al-Shawaf T Brinsden P The obstetric outcome of in vitrofertilization and embryo transfer in women with congenital uterine malformation. Am J Obstet Gynecol 1996;175:85–9. 8694080 10.1016/s0002-9378(96)70255-7

